# Vascular dysfunction in HFpEF: Potential role in the development, maintenance, and progression of the disease

**DOI:** 10.3389/fcvm.2022.1070935

**Published:** 2022-12-21

**Authors:** Andrea Saavedra-Alvarez, Katherine V. Pereyra, Camilo Toledo, Rodrigo Iturriaga, Rodrigo Del Rio

**Affiliations:** ^1^Laboratory of Cardiorespiratory Control, Department of Physiology, Pontificia Universidad Católica de Chile, Santiago, Chile; ^2^Centro de Excelencia en Biomedicina de Magallanes (CEBIMA), Universidad de Magallanes, Punta Arenas, Chile; ^3^Facultad de la Salud, Centro de Investigación en Fisiología y Medicina de Altura (MedAlt), Universidad de Antofagasta, Antofagasta, Chile

**Keywords:** inflammation, oxidative stress, heart failure, vascular dysfunction, preserved ejection fraction heart failure (HFpEF)

## Abstract

Heart failure with preserved ejection fraction (HFpEF) is a complex, heterogeneous disease characterized by autonomic imbalance, cardiac remodeling, and diastolic dysfunction. One feature that has recently been linked to the pathology is the presence of macrovascular and microvascular dysfunction. Indeed, vascular dysfunction directly affects the functionality of cardiomyocytes, leading to decreased dilatation capacity and increased cell rigidity, which are the outcomes of the progressive decline in myocardial function. The presence of an inflammatory condition in HFpEF produced by an increase in proinflammatory molecules and activation of immune cells (i.e., chronic low-grade inflammation) has been proposed to play a pivotal role in vascular remodeling and endothelial cell death, which may ultimately lead to increased arterial elastance, decreased myocardium perfusion, and decreased oxygen supply to the tissue. Despite this, the precise mechanism linking low-grade inflammation to vascular alterations in the setting of HFpEF is not completely known. However, the enhanced sympathetic vasomotor tone in HFpEF, which may result from inflammatory activation of the sympathetic nervous system, could contribute to orchestrate vascular dysfunction in the setting of HFpEF due to the exquisite sympathetic innervation of both the macro and microvasculature. Accordingly, the present brief review aims to discuss the main mechanisms that may be involved in the macro- and microvascular function impairment in HFpEF and the potential role of the sympathetic nervous system in vascular dysfunction.

## Introduction

Heart failure (HF) is a pathological condition affecting mainly the elderly population. A subcategory of this disease is HF with preserved ejection fraction (HFpEF), whose incidence has increased notably in recent years, particularly in the last two decades, from 48 to 57% compared with systolic HF (or reduced ejection fraction HF). Furthermore, HFpEF accounts for the death of 1 in 8 people over 65 years (1). Patients with HFpEF have a poor quality of life, high medical costs, and early death (2). Then, understanding the pathophysiology of HFpEF is relevant for future therapeutic strategies to improve HFpEF outcomes.

Patients with HFpEF display several comorbidities associated with cardiac and vascular disturbances, including but not limited to diabetes mellitus, obesity, pulmonary hypertension, coronary artery disease, chronic renal failure, and systemic inflammation (1), all of which contribute to endothelial dysfunction, cardiomyocyte hypertrophy, and cardiac fibrosis (2, 3). Furthermore, it has been described that autonomic imbalance, a hallmark of HF independent of its etiology (i.e., reduced or preserved EF), plays a key role in disease progression (4). Indeed, patients with HF showing sustained elevations in systemic circulating levels of catecholamines (i.e., norepinephrine) show higher mortality rates (5). Importantly, evidence indicates that the sympathetic nervous system (SNS) is critically influenced, at the central and peripheral levels, by the most relevant factors regulating vascular function, such as nitric oxide (NO), reactive oxygen species (ROS), endothelin 1 (ET-1), and the renin-angiotensin system (RAS). Then, a bidirectional and maladaptive relationship between endothelial function and hyperactivity of the SNS could play a role in short- and long-term vascular dysfunction in HFpEF. Indeed, autonomic imbalance in HFpEF increases sympathetic vasomotor tone (6). The latter results in increased excitatory sympathetic activity to blood vessels changing the balance between vasodilator and vasoconstrictor molecules that regulate endothelial cell function and therefore, cardiovascular integrity (7). In this review, we will focus on the main factors that may contribute to the development/maintenance of vascular cell dysfunction and their potential link to enhanced sympatho-vasomotor tone in the setting of HFpEF.

## Relevance of vascular dysfunction in HFpEF

Endothelium-dependent coronary microvascular dysfunction is present in approximately 30% of patients with HFpEF (8). In addition, more than 30% of patients with HFpEF display endothelium-independent dysfunction, reflected in significant reductions in coronary flow reserve (CFR) (8). Indeed, patients with HFpEF present vascular-ventricular uncoupling and stiffness, which is associated with decreased exercise capacity (9). Accordingly, acute increases in cardiac afterload, in the setting of arterial-ventricular stiffness, lead to increases in arterial blood pressure that impairs diastolic relaxation and increases filling pressures during exercise (10). The specific mechanisms associated with the changes in arterial elastance during HFpEF are not fully elucidated, but they have been associated with blood vessels alterations in the bioavailability and responses to vasoactive molecules such as ET-1 and NO (11, 12).

**Figure 1:**
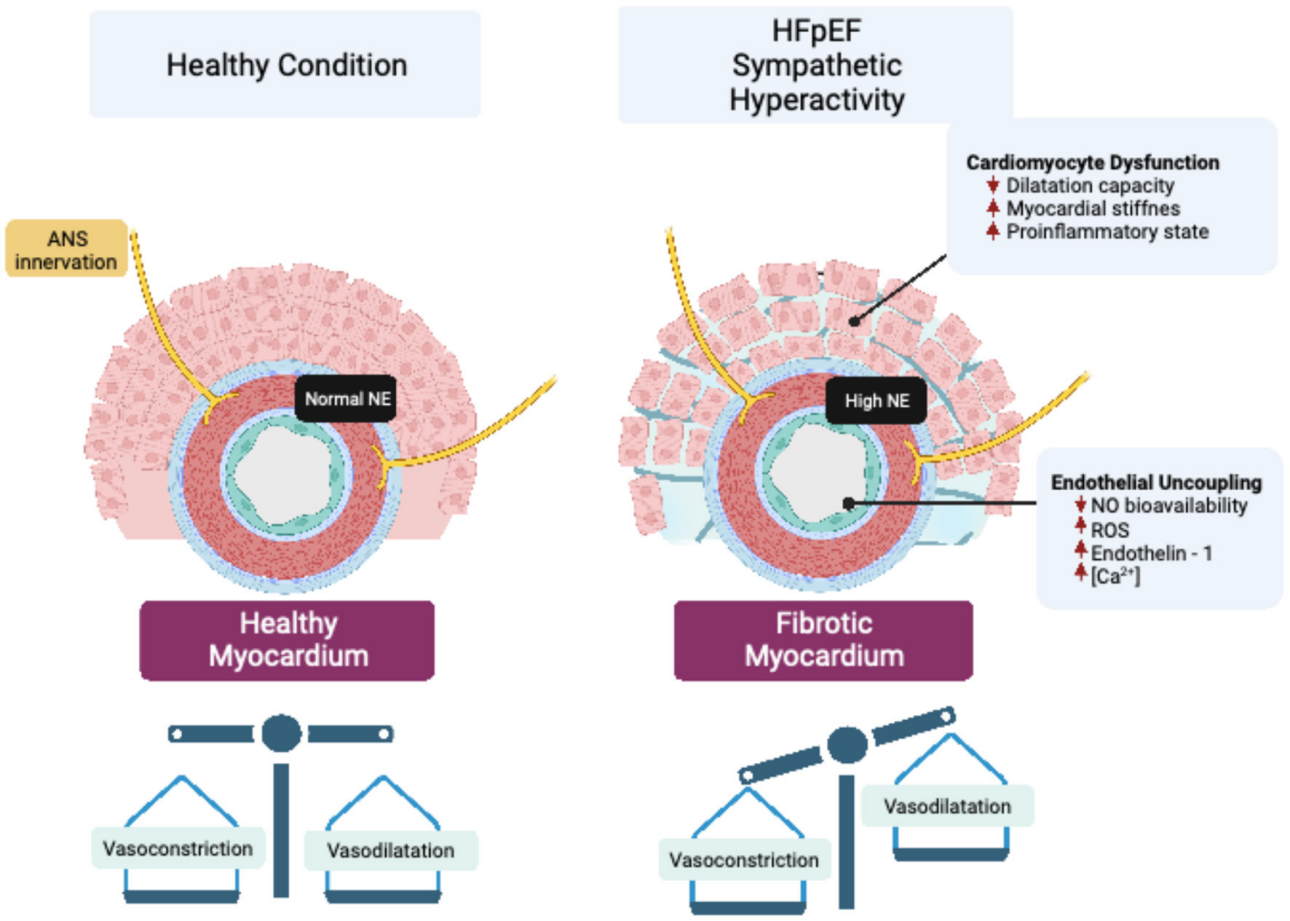
Vascular sympathetic neurotransmission and endothelial dysfunction in HFpEF. Enhanced sympathetic outflow led to increased release of norepinephrine (NE), which impair cardiovascular endothelial function by modifying peptides and signaling molecules that regulate perfusion to vascular beds. Endothelial uncoupling, in turn, can generate cardiomyocyte dysfunction that affects the structure and function of the heart through mechanisms associated with impaired myocardial dilatation capacity, stiffness, and inflammation.

In addition to systemic functional alterations in the vasculature, a reduction in myocardial microvascular density, called microvascular *rarefaction*, is observed in patients with HFpEF (13). Microvascular *rarefaction* contributes to cardiac perfusion failure by decreasing myocardial oxygen delivery in patients with HFpEF (14). Therefore, *rarefaction* of resistance vessels, including small arteries and arterioles, increases coronary microvascular resistance, resulting in reduced cardiac perfusion (15), which has been proposed as a pathogenic mechanism involved in the progressive decline in cardiac function in HFpEF (15). The precise mechanism(s) underpinning vascular *rarefaction* in HFpEF is still not completely known; however, due to the exquisite sympathetic regulation of blood vessels, and the fact that sympathoexcitation occurs in HFpEF, it is plausible that enhanced sympatho-vasomotor tone may play a role in vascular *rarefaction* by changing the vasoconstrictor to vasodilator balance in the vessel microenvironment (Figure 1).

To the best of our knowledge, there is no comprehensive literature providing mechanistic insights into macrovasculature changes in HFpEF. Macrovascular arterial stiffness results in an increase in pulse pressure and wave velocity, which impairs normal microvascular function (16). The latter is particularly relevant for the coronary and renal microvasculature since pathological alterations in pulse pressure and blood flow result in damage to the capillary network of these vascular territories (17). Indeed, coronary artery disease is considered an indicative sign of vascular dysfunction in patients with HFpEF (18). Arterial rarefaction and inadequate angiogenesis that take place during microvascular/macrovascular dysfunction may contribute to a decrease in oxygen supply to the myocardium (19). Accordingly, it has been proposed that left ventricular diastolic dysfunction in patients with HFpEF results from vascular alterations, with aortic stiffness and altered vascular endothelial function being fundamental characteristics of this process (20). Indeed, stiffness at the macrovasculature level is associated with ventricular decreases in elastance, leading to abnormal left arterio-ventricular crowning (21). Notably, ventricular stiffness occurs regardless of several comorbidities presented by patients with HFpEF (22). Besides the changes in vascular stiffness, studies in HFpEF also showed a decrease in brachial flow-mediated dilatation (FMD) and hyperemia, suggesting the presence of endothelial dysfunction at macrovascular/microvascular circulation. Lee et al. (23), proposed that macrovascular dysfunction is indeed a consequence of primary alterations at the microvascular level (23). This is in line with a previous report showing the presence of endothelial dysfunction at the microvasculature with no overt signs of vascular dysfunction in conductance vessels in experimental HFpEF models (24). Together, current evidence supports the role of microvascular/macrovascular alterations in the progression of heart disease. Whether changes/adaptations in the microvasculature/macrovasculature are a cause or consequence to support the failing heart (in the setting of heart failure) remains to be determined.

## Autonomic imbalance and vascular dysfunction: Main mechanisms involved

The endothelium is a highly dynamic layer that works as a barrier that separates the blood from the extravascular tissue and interacts with other cell types contributing to the physiological and homeostatic regulation of blood vessel function (25). In addition, the endothelium prevents the aggregation and adhesion of platelets and leukocytes, inhibits the proliferation of smooth muscle cells (SMC), regulates vascular tone, and plays a protective role against mechanical stimuli such as pressure or frictional stress, through the release of vasoactive substances. This is critical for the maintenance of adequate organ/tissue perfusion (26, 27). While endothelial cells (EC) are located in the most internal layer of blood vessels, SMCs are located in the medial layer and constitute the contractile elements of blood vessels, contributing to the regulation of blood vessel tone, blood pressure, and circulation (28). Then, the correct function of SMC and EC is important for vascular health since both manage vasomotor tone and vasculature integrity.

Both arms of the autonomic nervous system (ANS) (i.e., sympathetic and parasympathetic) innervate blood vessel walls and regulate wall tension (29–31). SMCs at the muscular layer of blood vessel walls receive adrenergic and cholinergic nerve projections from sympathetic and parasympathetic innervation, while ECs do not present a direct neural innervation from the ANS (29, 31). The vascular SMC layer encompasses several ANS nerve terminals. Indeed, SMC constitutively expresses β-adrenergic receptors, which modulate vasodilatation, and α_1_/α_2_-adrenergic receptors, which modulate vasoconstriction (29, 31). In addition, parasympathetic stimulation of muscarinic receptors within SMCs also results in blood vessel contraction. Despite not being directly innervated by the sympathetic-adrenergic system, ECs also constitutively express both β-adrenoreceptors and α_2_-adrenoreceptors. While the effects of β-adrenoreceptors stimulation on EC function remain unknown, the activation of α_2_-adrenoreceptors leads to the release of vasoactive molecules such as nitric oxide (NO), which acting at SMC induces cell relaxation resulting in blood vessel vasodilation (29, 31). Besides the fine regulation of vascular function by the ANS, how autonomic imbalance could affect vasculature integrity by modulating mechanisms associated with vasoconstriction/relaxation and the vasculature environment is not completely understood, and much less is known about these mechanisms in the pathological setting of HFpEF. In this review, we discussed the potential mechanism of vascular dysfunction in HFpEF and its relation to autonomic imbalance.

### Nitric oxide signaling and oxidative stress

The role of vascular NO is essential for vasodilation, inhibition of platelet aggregation, and protection of the integrity of the endothelial layer given its anti-inflammatory, proangiogenic, anti-apoptotic, and anti-fibrotic properties, reducing vascular inflammation and atherosclerosis (32, 33). At the major circulation, NO diffuses into platelets and SMC from EC, which stimulates soluble guanylate cyclase (sGC) and activates the cyclic GMP (cGMP) pathway to induce calcium release from the sarcoplasmic reticulum (SCR) in SMC, preventing platelet aggregation and producing vasodilation, respectively. At the level of cardiac microcirculation, NO can diffuse into cardiomyocytes from adjacent coronary vasculature, modulating cardiac function (7). In addition, NO signaling is involved in tissue repair by mediating the mobilization of stem and progenitor cells (34). In HFpEF, endothelial dysfunction has been linked to decreased production of cGMP and reduced activity of protein kinase G (PKG) and the L-arginine-NO synthetic pathway. Therefore, mechanisms for vasodilation are likely to be impaired in patients with HFpEF. Interestingly, vascular endothelial dysfunction in the heart shared similar mechanisms compared to those found in the systemic circulation, being alterations in sGC-cGMP signaling a common pathway affected at both levels during the progression HFpEF. More importantly, alterations in the sGC-cGMP-PKG pathway in HFpEF promote functional impairment in cardiomyocytes, as evidenced by delayed myocardial relaxation, increased myocardial stiffness, cardiac hypertrophy, and interstitial fibrosis (35). Therefore, direct interventions targeting the NO/cGMP/PKG pathway have been proposed as novel therapeutics to improve both vascular and cardiac function in HFpEF (36, 37).

How autonomic imbalance, a hallmark pathophysiological condition found in experimental and human HFpEF, affects vascular NO production is still not known. Endothelial β_2_-adrenergic receptors stimulate NO synthesis by the activation of endothelial nitric oxide synthase isoform (eNOS) (32). Interestingly, overexpression or chronic activation of eNOS could be maladaptive since marked increases in intracellular oxidative stress have been reported following eNOS overexpression (38, 39). Furthermore, chronic β-adrenoreceptor activation exacerbates eNOS activity and upregulates eNOS gene expression, favoring superoxide anion generation and vascular dysfunction through reductions in NO bioavailability (38, 40). Indeed, oxygen free radicals rapidly react with NO to form reactive nitrogen species, which are known to promote a prothrombotic and proinflammatory niche within blood vessels (12, 41). Notably, the relevance of reduced NO bioavailability and increased oxidative stress to promote HFpEF pathophysiology has been demonstrated in experimental HF in which concomitant metabolic and vascular stress in mice (high-fat diet and constitutive NOS inhibition using N(omega)-nitro-L-arginine methyl ester) recapitulated the cardiovascular features of human HFpEF (12, 26). Therefore, it is plausible that hyperactivation of the sympathetic nervous system in HFpEF may lead to decreases in NO bioavailability by promoting the formation of reactive nitrogen species within blood vessels. Further investigation is needed to fully determine the contribution of enhanced sympathetic activity on NO and vascular alterations in HFpEF. In addition, HFpEF increases ROS levels and/or antioxidant enzyme suppression, leading to cardiac and endothelial dysfunction. The different risk factors for HFpEF stimulate the production of ROS (42–44). Oxidative stress by their side increases levels of hydrogen peroxide and reactive oxidative metabolites, uncoupled endothelial nitric oxide synthase, endothelial NADPH oxidase 2 (NOX2) expression, and reduced NO levels indicate the presence of myocardial oxidative stress in patients with HFpEF (45). Beyond oxidation, inhibition of NO production can reduce NO bioavailability, for example, through AGE-induced elevation of asymmetric levels of ADMA (dimethyl L-arginine), an inhibitor of eNOS (endothelial NOS), which contributes to endothelium-dependent dysfunction associated with poorer HFpEF prognosis (46). Also, autonomic dysfunction characterized by chronic activation of the SNS might contribute to oxidative stress at the EC level. Previous reports showed high contractile activity in β_2_-adrenoreceptor deficient mice, and this loss of function can trigger ROS-mediated NO impairment (47). Thus, a lack of β_2_ receptors increases oxidative stress in the β_2_-KO mice arteries, and this change the vasoconstrictor response to phenylephrine. In addition, the above evidence suggests a crucial link between adrenergic pathways, oxidative stress, and NO bioavailability in the vasculature (47, 48). Interestingly, patients with HFpEF display not only impaired catecholamine sensitivity and β-adrenoreceptor density at the cardiac level (49, 50) but also display impaired chronotropic and vasodilatation response to exercise (51), suggesting possible desensitization of adrenergic signaling at the cardiac and vascular level. Overall, heightened SNS activity in the setting of HFpEF might contribute to creating a vicious cycle that promotes and maintains vascular dysfunction.

### Inflammatory status

Risk factors in HF, such as diabetes mellitus, aging, and hypertension, among others, trigger systemic low-grade inflammation, characterized by chronic elevations in circulating immune cells, proinflammatory cytokines, and increased expression of endothelial adhesion molecules, such as vascular and intercellular cell adhesion molecules-1 (ICAM-1 and VCAM-1), and the corresponding ligands of circulating leukocytes, increasing myocardial infiltration of CD45+ and CD3+ T-lymphocytes (52). The latter further promotes the infiltration of leukocytes, especially monocytes, into the myocardial tissue, increasing the release of transforming growth factor beta (TGF-β), which ultimately leads to extracellular matrix remodeling and fibrosis (41, 43). Importantly, it has been reported that flow-mediated dilation (FMD) and reactive hyperemic index (RH) are reduced in patients with HFpEF (45), which is closely associated with elevations in inflammatory markers, such as CRP, IL-6, TNF-α, IL-1β, and NFG15 (53, 54). The increase in the inflammatory status leads to coronary microvascular endothelial dysfunction and further increases in inflammatory cytokines (55) partially mediated by the activation of the nuclear factor-kappa B (NFkB) signaling pathway (43). Thus, microvascular dysfunction is proposed to be the central mediator connecting systemic low-grade inflammation with myocardial dysfunction and remodeling in the setting of HFpEF (35).

### Calcium signaling

Chronic elevation of catecholamines in HF, such as epinephrine and norepinephrine, is a hallmark and strong predictor of mortality in patients with HF (56, 57). Catecholamines activate the adenyl cyclase (AC)-cAMP-PKA pathway, leading to IP_3_R1 activation and in consequence IP_3_ signal to increased Ca^2+^ release and vascular tone in VSMCs during HF (58). Also, it has been found that BK potassium channels, which contribute to VSMC hyperpolarization, are downregulated in HF, promoting vasoconstriction, and synergizing with IP_3_R1 for elevations in cytosolic [Ca^2+^] (59). Since mRNA and protein levels of inositol 1,4,5 phosphate receptor 1 (IP_3_R1) are upregulated in HF and increased receptor phosphorylation in HF, it has been suggested that IP_3_R1 may play an important role in Ca^2+^ regulation in VSMC (60, 61). However, little is known about the contribution of intracellular calcium (Ca^2+^) mishandling in the vasculature and subsequent acceleration of cardiac remodeling and progression of HFpEF (58). Nevertheless, alterations in the expression and function of proteins that handle Ca^2+^ and a maladaptive redistribution of intracellular calcium have been described in HF (62). Some of these proteins are RyR2, Serca2a, Na^+^-Ca^2+^ exchanger (NCX), and transient receptor potential cation channels (TRPC) (63). For RyR2, there is evidence of PKA-dependent hyperphosphorylation (in S2808), causing channel dissociation, increasing Ca^2+^ leakage from the SR, decreasing Ca^2+^ transients, changing spontaneous Ca^2+^ release events, and altering cytosolic Ca^2+^ management (64). In addition, Serca2a is downregulated in HFpEF, then Ca^2+^ reuptake toward the SR affecting both active and passive cardiovascular functions (65). In addition, increased activity of NCX in HFpEF has also been described (66). Finally, the TRPC channels that participate in the entry of Ca^2+^ from the extracellular medium that allows the increase of Ca^2+^ reservoirs into the SR are increased in HFpEF, possibly as an adaptive mechanism due to a decrease in Ca^2+^ reserves in the SR (67). In addition, increased myosin heavy chain phosphorylation has also been found in the arteries of patients with HF and mice (68). The latter has been linked to VSMC remodeling and has been associated with alterations in VSMC Ca^2+^ handling (69). Therefore, alterations in the management of intracellular Ca^2+^ in the vasculature in HF may play an important role not only in vascular cell function but also in the adverse remodeling of several vascular compartments.

## Conclusion

Little is known about the role of macro- and microvascular alterations during the onset, development, and progression of HFpEF. However, it is highly likely that vascular rarefaction takes place during the onset, maintenance and/or progression of HFpEF resulting in increases in microvascular resistance, reductions in tissue perfusion, and activation of vasomotor sympathetic fibers that ultimately create a feed-forward mechanism that promotes the further deterioration of vascular function by shifting the balance between vasoconstriction and vasodilation. On the contrary, proinflammatory and pro-oxidative molecules have been associated with the etiology of the disease. At the microvascular level, the decrease in the bioavailability of NO, alterations in the sGC-cGMP-PKG pathway, accumulation of ROS, and chronic low-grade inflammation are the main actions involved in the alteration of vascular function both at the systemic circulation and in the coronary territory, promoting a functional decrease in cardiomyocytes, evidenced by delayed myocardial relaxation, increased myocardial stiffness, cardiac hypertrophy, and interstitial fibrosis. The latter may have fundamental implications for the progressive decline in cardiac function during HFpEF.

To date, there are only preventive and palliative actions to deal with HFpEF, such as exercise and a healthy lifestyle, which do not imply a remission of the disease. In this article, several molecular candidates rise as potential therapeutic targets to improve both vascular and cardiac functions in HFpEF, including but not limited to NO metabolic pathway, IP3R signaling, adrenergic pathways, and reduction of oxidative stress and vascular inflammation.

## Author contributions

AS-A wrote the first draft. KP, CT, RI, and RDR contributed to manuscript formulation and revision. All authors have read and approved the final manuscript.
